# Annotation of Two Large Contiguous Regions from the *Haemonchus contortus* Genome Using RNA-seq and Comparative Analysis with *Caenorhabditis elegans*

**DOI:** 10.1371/journal.pone.0023216

**Published:** 2011-08-15

**Authors:** Roz Laing, Martin Hunt, Anna V. Protasio, Gary Saunders, Karen Mungall, Steven Laing, Frank Jackson, Michael Quail, Robin Beech, Matthew Berriman, John S. Gilleard

**Affiliations:** 1 Welcome Trust Sanger Institute, Wellcome Trust Genome Campus, Hinxton, Cambridge, United Kingdom; 2 Faculty of Veterinary Medicine, University of Glasgow, Glasgow, Strathclyde, United Kingdom; 3 Genome Sciences Centre, BC Cancer Agency, Vancouver, British Columbia, Canada; 4 Moredun Research Institute, Pentlands Science Park, Bush Loan, Penicuik, United Kingdom; 5 Institute of Parasitology, McGill University, Ste Anne de Bellevue, Quebec, Canada; 6 Faculty of Veterinary Medicine, University of Calgary, Calgary, Alberta, Canada; New England Biolabs, United States of America

## Abstract

The genomes of numerous parasitic nematodes are currently being sequenced, but their complexity and size, together with high levels of intra-specific sequence variation and a lack of reference genomes, makes their assembly and annotation a challenging task. *Haemonchus contortus* is an economically significant parasite of livestock that is widely used for basic research as well as for vaccine development and drug discovery. It is one of many medically and economically important parasites within the strongylid nematode group. This group of parasites has the closest phylogenetic relationship with the model organism *Caenorhabditis elegans*, making comparative analysis a potentially powerful tool for genome annotation and functional studies. To investigate this hypothesis, we sequenced two contiguous fragments from the *H. contortus* genome and undertook detailed annotation and comparative analysis with *C. elegans*. The adult *H. contortus* transcriptome was sequenced using an Illumina platform and RNA-seq was used to annotate a 409 kb overlapping BAC tiling path relating to the X chromosome and a 181 kb BAC insert relating to chromosome I. In total, 40 genes and 12 putative transposable elements were identified. 97.5% of the annotated genes had detectable homologues in *C. elegans* of which 60% had putative orthologues, significantly higher than previous analyses based on EST analysis. Gene density appears to be less in *H. contortus* than in *C. elegans*, with annotated *H. contortus* genes being an average of two-to-three times larger than their putative *C. elegans* orthologues due to a greater intron number and size. Synteny appears high but gene order is generally poorly conserved, although areas of conserved microsynteny are apparent. *C. elegans* operons appear to be partially conserved in *H. contortus*. Our findings suggest that a combination of RNA-seq and comparative analysis with *C. elegans* is a powerful approach for the annotation and analysis of strongylid nematode genomes.

## Introduction

*H. contortus* is a parasitic nematode of small ruminants of major economic importance. It is also one of the most experimentally tractable parasitic nematodes and is widely used as a model parasite for studies on basic parasite biology [1], [2], vaccine development [3], [4], drug discovery [5] and anthelmintic resistance [6]–[8]. Comparative genomic analysis between *H. contortus* and *C. elegans* is essential to explore the extent and limitations in which *C. elegans* can be used as a model for the strongylids and will have reciprocal benefits for research on this widely-studied nematode.

There has been a recent explosion in the number of nematode genomes being sequenced. However, experience from other organisms shows that draft genome assemblies vary enormously in quality and this creates huge problems for research communities and significantly reduces the value of the resources [9]. Hence, the major challenge facing parasitic nematode genomics is not genome sequencing per se, but assembly and annotation. The production of a high quality finished genome sequence for *H. contortus* is an important aim as it will provide a reference genome for many parasitic nematodes in the strongylid nematode group. These include some of the most important “neglected tropical diseases” of humans and economically important parasites of livestock, many of which are currently being sequenced to varying levels of completion (e.g. http://www.sanger.ac.uk/resources/downloads/helminths/ and http://www.nematode.net/). However, the assembly of the *H. contortus* genome is a major challenge, predominantly due to genome size and sequence polymorphism; factors likely to be common to many other nematode genomes. The latest assembly of ∼4 Gb capillary, 454 and Illumina sequencing has generated a 393 Mb assembly, with a contig N50 of 6447 bp and mean length of 3520 bp. The contig sizes are not sufficiently large to allow a detailed global analysis of genome organisation and gene structure or to undertake a comprehensive comparative analysis with *C. elegans*. Consequently, to investigate the utility of RNA-seq together with comparative analysis with *C. elegans* for strongylid nematode genome annotation, we have undertaken detailed annotation of two large and manually finished contiguous regions (409 kb and 181 kb). We have used Illumina technology to sequence the adult *H. contortus* transcriptome and used the RNA-seq data to annotate the genomic sequences at a single nucleotide level. Here we present the results of this annotation, along with a comparative analysis of *H. contortus* and *C. elegans* gene structure and genome organisation.

## Results

Illumina technology was used to sequence the adult *H. contortus* MHco3 (ISE) isolate transcriptome. 38 million 76 bp reads were generated and mapped onto genomic sequence to guide annotation of a 409 kb overlapping BAC tiling path (X-linked contig) and a 181 kb BAC insert sequence (BAC BH4E20). In total, the 590 kb genomic sequence had a GC content of 43%, with a 47.6% and 46.3% GC content for the identified coding sequence in the X-linked contig and BAC BH4E20 respectively. These figures were slightly higher than the *C. elegans* genome GC content of 35.4% and exon GC content of 42.7% [10].

### Gene density is lower on the *H. contortus* contigs than on equivalent regions of the *C. elegans* genome

A total of 52 transcripts were identified across the two contigs. 37 were coding sequences mapped with RNA-seq: 16 transcripts on the X-linked contig and 21 transcripts on BAC BH4E20. An additional 14 coding sequences with a low coverage of mapped reads were predicted on the X-linked contig with the gene prediction software Genefinder. One β-tubulin gene, *hc-18h7-1*, was annotated from sequenced cDNA. 12 of the 52 transcripts were identified as transposable elements (TEs), so were excluded from the analysis of gene density and structure and are discussed later.

Thus, 40 putative genes were identified in a total of 590 kb genomic sequence, which is a density of one gene per 14.75 kb. The genome average for *C. elegans* is one gene per 5 kb [10]. 23 of these transcripts were identified on the 409 kb X-linked contig, which is a density of one gene per 17.78 kb. The average gene density on the X chromosome in *C. elegans* is one gene per 6.54 kb [10]. 17 transcripts were identified on the 181 kb BAC insert BH4E20, which is a density of one gene per 10.65 kb, relative to a *C. elegans* average of one gene per 4.77–5.06 kb on chromosome I, the range reflecting a higher density in the central cluster region than in the arms [10].

### Comparison of orthologous genes suggests gene size is significantly larger in *H. contortus* than *C. elegans*

The conceptual translations of 22 transcripts were most similar to *C. elegans* proteins, eleven transcripts were most similar to *C. briggsae* predicted proteins and three were most similar to *Brugia malayi* proteins, based on current NCBI databases. The conceptual translations of three transcripts were most similar to proteins outside *Nematoda*. One transcript, *hc-bh4e20-1*, predicted to encode a P-glycoprotein (PGP) shared most identity with a published *H. contortus* PGP polypeptide (accession number AAC38987), but was also highly similar to *C. elegans PGP-2*.

Homologous proteins (BLASTp, Expect-value (E)>1e-10) were identifiable in *C. elegans* for all three predicted polypeptides with most identity to *B. malayi* proteins. For the three genes with a closest match outside *Nematoda*, two encoded proteins that were highly conserved in many species (*hc-13c1-3* encoded a putative RNA-binding protein and *hc-bh4e20-15* encoded a putative high mobility group protein) and both had homology (BLASTp, E>1e-10) to proteins in *C. elegans*. The third gene, *hc-13c1-2*, encoded a conserved F-box domain yet shared little identity with any sequence in NCBI databases.

**Figure 1:**
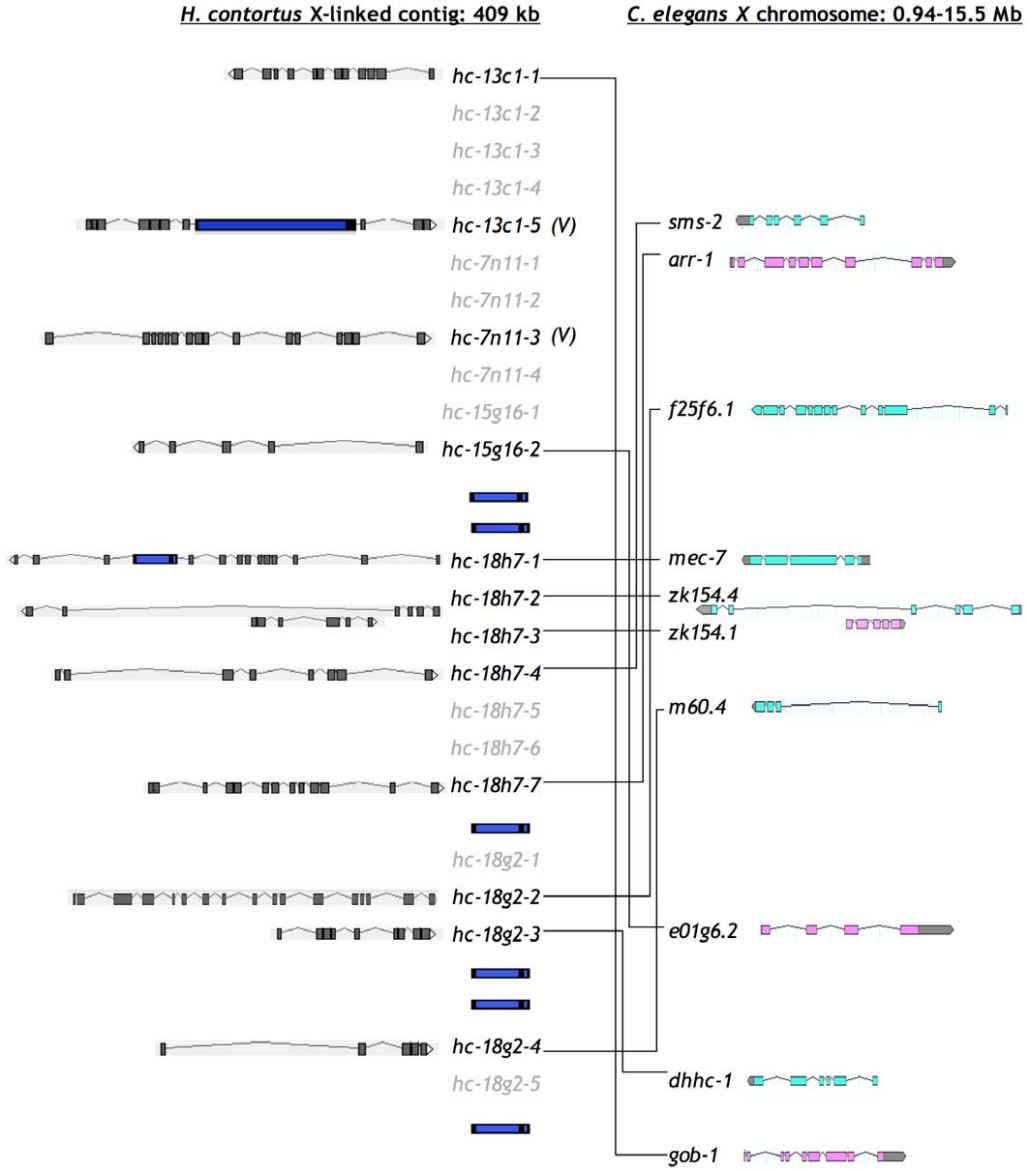
Conserved microsynteny between *H. contortus* X-linked contig and *C. elegans* X chromosome. Putative orthologues are in black type, genes with no clear orthologues are in grey type and transposon insertions are shown as dark blue ORFs. Colinearity is maintained in *H. contortus* orthologues of *C. elegans mec-7*, *zk154.4* and *zk154.1*. *hc-13c1-5* and *hc-7n11-3* are orthologous with genes on *C. elegans* chromosome V.

**Figure 2:**
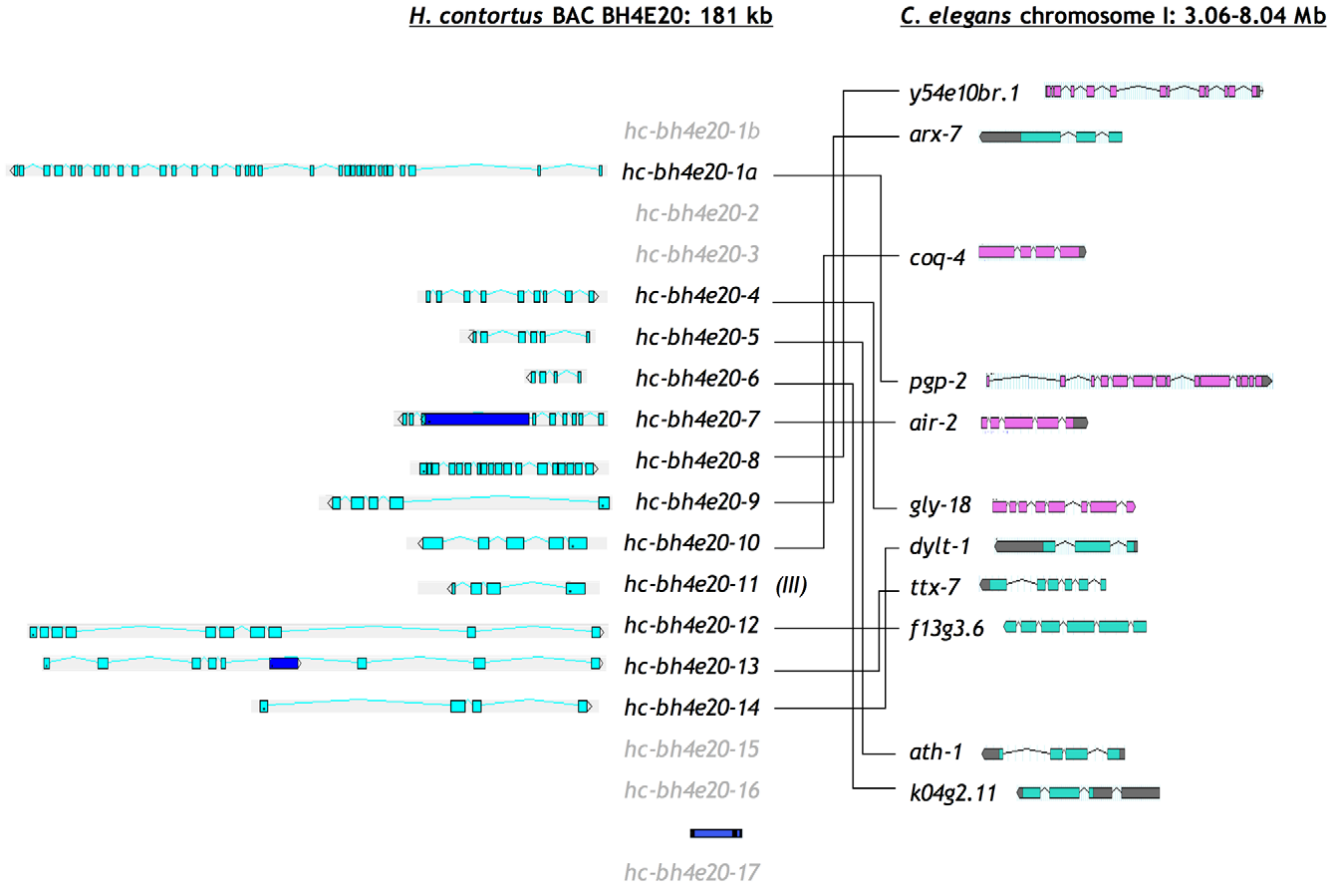
Conserved microsynteny between *H. contortus* BAC BH4E20 and *C. elegans* chromosome I. Putative orthologues are in black type, genes with no clear orthologues are in grey type and transposon insertions are shown as dark blue ORFs. Colinearity is maintained in *H. contortus* orthologues of *C. elegans y54e10br.1* and *arx-7*, orthologues of *C. elegans dylt-1*, *ttx-7* and *f13g3.6* and orthologues of *C. elegans ath-1* and *k04g2.11*. The last two gene sets are in operons in *C. elegans*. *hc-bh4e20-11* is orthologous with *f53a3.7* on *C. elegans* chromosome III.

To assess the extent of conservation of gene structure between *H. contortus* and *C. elegans*, a subset of 24 putative orthologues of *C. elegans* genes were identified in the annotated parasite sequence. The inherent risk in inferring an orthologous or paralogous relationship between *C. elegans* genes and those from the incomplete *H. contortus* genome is that closer relatives may be identified when the parasite genome is fully sequenced. With this caveat in mind, 21 *H. contortus* putative orthologues of *C. elegans* genes were identified using sequence similarity criteria (see Materials and Methods), while a further three were identified through conserved microsynteny. Gene *hc-18h7-3* lies within the fourth intron of gene *hc-18h7-2* on the complementary strand, an identical relationship to their closest matched genes, *zk154.1* and *zk154.4*, in *C. elegans* (Figure 1). Gene *hc-bh4e20-6* lies on the same strand and directly upstream of the orthologue of *C. elegans ath-1*, and shares most homology with *k04g2.11*, the corresponding upstream gene in *C. elegans* (Figure 2). This conservative subset of 24 orthologous genes was used to directly compare *H. contortus* and *C. elegans* gene structure (Table 1).

**Table 1:** Subset of 24 *C. elegans* and *H. contortus* putative orthologues.

*H. contortus*				*C. elegans*					
Gene	Unspliced	Spliced	Introns	Gene	Unspliced	Spliced	Introns	Chromosome	BLASTp
*hc-13c1-1*	4695	1437	11	*gob-1*	3408	1407	8	X	8.00E-136
*hc-13c1-5*	4392	1200	10	*folt-1*	1566	1233	4	V	5.00E-105
*hc-7n11-3*	8243	1596	15	*klc-2*	2402	1623	4	V	0
*hc-15g16-2*	6796	636	4	*e01g6.2*	1898	615	3	X	3.00E-41
*hc-18h7-1*	10663	1326	12	*mec-7*	1596	1326	4	X	0
*hc-18h7-2*	7731	642	5	*zk154.4*	5132	612	5	X	5.00E-17
*hc-18h7-3*	3341	912	5	*zk154.1*	895	630	4	X	1.00E-78
*hc-18h7-4*	8954	1059	7	*sms-2*	3616	1008	5	X	6.00E-149
*hc-18h7-7*	6811	1443	11	*arr-1*	3230	1308	9	X	8.00E-180
*hc-18g2-2*	9409	2667	16	*f25f6.1*	5991	2340	12	X	1.00E-135
*hc-18g2-3*	3556	879	8	*dhhc-1*	2218	888	5	X	1.00E-92
*hc-18g2-4*	5360	486	4	*m60.4*	3871	489	3	X	1.00E-59
*hc-bh4e20-1a*	18110	3825	32	*pgp-2*	9012	3819	13	I	0
*hc-bh4e20-4*	4766	1035	8	*gly-18*	2048	1323	7	I	7.00E-78
*hc-bh4e20-5*	3295	660	5	*ath-1*	1656	672	3	I	7.00E-83
*hc-bh4e20-6*	1371	357	3	*k04g2.11*	352	258	2	I	3.00E-08
*hc-bh4e20-7*	2799	846	8	*air-2*	1206	918	4	I	4.00E-87
*hc-bh4e20-8*	4921	2646	17	*y54e10br.1*	9937	2739	12	I	0
*hc-bh4e20-9*	2633	459	4	*arx-7*	645	459	2	I	8.00E-56
*hc-bh4e20-10*	1558	708	4	*coq-4*	844	696	3	I	1.00E-87
*hc-bh4e20-11*	1258	396	3	*f53a3.7*	485	360	2	III	7.00E-26
*hc-bh4e20-12*	6998	1173	9	*f13g3.6*	1343	1074	5	I	4.00E-92
*hc-bh4e20-13*	6517	858	7	*ttx-7*	1805	858	5	I	3.00E-118
*hc-bh4e20-14*	3107	348	3	*dylt-1*	543	321	2	I	1.00E-31
**Average**	5720.17	1149.75	8.79		2737.46	1124.00	5.25		
**Median**	4843.50	895.50	7.5		1851.5	903	4		

Sizes in nucleotides or amino acids as appropriate. Unspliced length is genomic sequence from ATG to terminal stop codon (does not include 5′ or 3′ UTR). Spliced length is predicted protein coding sequence only.

The parasite genes had a similar spliced transcript size to their homologues in *C. elegans*, but unspliced transcript size was invariably larger, which was a function of both a greater number of introns and a larger intron size in *H. contortus* (Table 1). Average unspliced transcript length was 5.72 kb (median 4.84 kb) compared to an average of 2.74 kb (median 1.85 kb) for the orthologous gene set in *C. elegans* and an average of 2.5 kb (median 1.91 kb) in the *C. elegans* genome [11], [12]. Predicted UTRs were excluded in the above calculations and any transcribed sequences located within an intron (e.g. putative transposable elements and genes *hc-18h7-3* and *zk154.1*) were subtracted from the unspliced transcript size.

The average number of introns was 8.79 per gene (median 7.5) in *H. contortus* compared to an average of 5.25 introns per gene (median 4) in the orthologous gene set in *C. elegans* and a genome average of 4 per gene (median 5) in the model worm [12], [13]. The average intron size in this *H. contortus* subset was 520 bp compared to an average intron size of 360 bp in the *C. elegans* orthologues. The genome average intron size for *C. elegans* is 466.6 bp, although this is skewed by a small number of very large introns, giving a more representative median of 65 bp [13], [14]. The average for *C. elegans* in this subset was inflated by a large first intron in gene *m60.4*, which is conserved in the orthologous gene, *hc-18g2-4*, in *H. contortus* (Figure 1).

### Synteny/Colinearity between *H. contortus* and *C. elegans*

A previous study showed that the 409 kb contig was X-linked in *H. contortus* based on male/female genotyping and inheritance of a panel of six microsatellites distributed along the contig [15]. Of the 12 *H. contortus* genes on this contig with clear putative orthologues in *C. elegans*, ten had orthologues located on the *C. elegans* X chromosome and the remaining two had orthologues on *C. elegans* chromosome V (Figure 1). Although these ten genes were located within a small (409 kb) region of the *H. contortus* X chromosome, the orthologues were spread over more than 14 Mb of the *C. elegans* X chromosome, with generally poor conservation of long range gene order and polarity (Figure 1). However, a single region of conserved microsynteny was apparent between genes *hc-18h7-1*, *hc-18h7-2* and *hc-18h7-3* in *H. contortus* and *mec-7*, *zk154.1* and *zk154.4* on the X chromosome in *C. elegans*.

For BAC BH4E20, of the 12 predicted polypeptides that had clear orthologues in *C. elegans*, 11 of these were encoded on *C. elegans* chromosome I and one, HC-BH4E20-11, was encoded on *C. elegans* chromosome III (Figure 2). Again, these orthologues were scattered across a large region of *C. elegans* chromosome I despite being located on this relatively small region (181 kb) of the *H. contortus* genome with little long range synteny or conservation of polarity. However, three regions of microsynteny were apparent: *H. contortus* genes *hc-bh4e20-8* and *hc-bh4e20-9* had a conserved relationship relative to the orthologous *C. elegans* genes *y54e10br.1* and *arx-7*; as did *H. contortus* genes *hc-bh4e20-12*, *hc-bh4e20-13* and *hc-bh4e20-14* to *C. elegans* genes *dylt-1*, *ttx-7* and *f13g3.6*; and *H. contortus* genes *hc-bh4e20-5* and *hc-bh4e20-6* to *C. elegans* genes *ath-1 and k04g2.11*.

In order to investigate whether the syntenic relationships of the two large *H. contortus* contigs with *C. elegans* sequence was likely to be representative across the genome, a survey analysis of BAC end derived sequence was undertaken. The *H. contortus* BAC end database contains 20,828 sequences of an average of 760 bp, corresponding to each end of 10,414 BAC inserts. This dataset was used for a BLASTx search of *C. elegans* Wormpep and the locus of the best-matched *C. elegans* gene for every hit with P<0.01 was recorded. 233 BAC inserts had matches to *C. elegans* proteins at both ends using these criteria (Supplementary Information, Table 1). 118 of these BAC end pairs (50.64%) hit *C. elegans* genes on the same chromosome compared to 16.67% that would be expected by chance if there was no conservation of chromosomal location. Although the BLASTx matches cannot be claimed to represent definite orthologous pairs, a random selection of genes would not be expected to yield a higher linkage estimate. This suggests there is a high degree of chromosomal synteny between *H. contortus* and *C. elegans* genomes, consistent with our analysis of the two large contigs.

### Trans-splicing of the annotated *H. contortus* genes

Around 15% of *C. elegans* genes are in operons. Although both SL1 and SL2 trans-splicing has been described in *H. contortus*, its frequency of occurrence and relationship to operon structure is unknown [16]–[18]. In order to investigate which genes on the two annotated contigs were trans-spliced to SL1 and SL2, transcriptome reads containing SL1 and SL2 sequences were identified and trimmed to remove the spliced leader sequence, then mapped against the annotated sequence of the X-linked contig and BAC BH4E20 (SL RNA-seq). The results are shown in Table 2. No SL RNA-seq reads were detected for 26 genes. Of the remainder, ten genes were trans-spliced with SL1, two genes were trans-spliced with SL2 sequences and two genes were trans-spliced with SL1 and SL2 sequences. Alternative start codons were identified for six genes and the same spliced leader was used for alternative transcripts of the same gene (Table 2).

**Table 2:** Trans-splicing.

Gene	Spliced Leader	Alternative Start Codon	Intergenic Distance if SL2 Trans-spliced
*hc-13c1-1*	SL1	first codon exon 2 (SL1)	
*hc-13c1-4*			
*hc-13c1-5*			
*hc-18g2-2*			
*hc-18g2-3*	SL1	mid exon 2 (SL1)	
*hc-18g2-4*	SL1		
*hc-18h7-1*			
*hc-18h7-2*			
*hc-18h7-3*			
*hc-18h7-4*			
*hc-18h7-7*	SL1	mid exon 2 (SL1)	
*hc-7n11-3*			
*hc-18h7-6*			
*hc-13c1-2*			
*hc-13c1-3*	SL1		
*hc-15g16-1*	SL1		
*hc-15g16-2*	SL2		no upstream gene for >35 kb
*hc-18h7-5*			
*hc-7n11-1*			
*hc-7n11-2*	SL1		
*hc-7n11-4*			
*hc-18g2-1*			
*hc-18g2-5*			
*hc-bh4e20-1a*			
*hc-bh4e20-2*			
*hc-bh4e20-3*	SL2		all upstream genes on opposite strand for >47 kb
*hc-bh4e20-4*			
*hc-bh4e20-5*	SL1/SL2	first codon exon 2 (SL1/SL2)	1538 bp downstream from *hc-bh4e20-6* on same strand
*hc-bh4e20-6*			
*hc-bh4e20-7*			
*hc-bh4e20-8*	SL1		
*hc-bh4e20-9*			
*hc-bh4e20-10*			
*hc-bh4e20-11*			
*hc-bh4e20-12*			
*hc-bh4e20-13*			
*hc-bh4e20-14*	SL1/SL2		403 bp downstream from *hc-bh4e20-13* on same strand
*hc-bh4e20-15*	SL1	mid exon 2 (SL1)	
*hc-bh4e20-16*	SL1		
*hc-bh4e20-17*			

The spliced leader trans-spliced to each transcript was identified using SL-trimmed RNA-seq data where available. Alternative start codons were identified for six transcripts and the same spliced leader was used for alternative transcripts of the same gene. Intergenic distance of the nearest upstream gene is shown for the four sequences trans-spliced to SL2.

### Operon structure is partially conserved between *H. contortus* and *C. elegans*

Of the 12 *H. contortus* genes on the X-linked contig that had orthologues in *C. elegans*, only one, *hc-13c1-1*, had a *C. elegans* orthologue that was in an operon. This was *gob-1*, which is in operon CEOPX136 and is transcribed with the downstream gene *h13n06.4*. However, the *H. contortus gob-1* orthologue, *hc-13c1-1*, has no identifiable gene within the 10 kb available downstream sequence based on BLASTx similarity, RNA-seq or Genefinder predictions suggesting this gene is not in an operon in *H. contortus*. A reciprocal BLAST search with the H13N06.4 polypeptide hit no sequence within the BAC contig database as expected, but did identify homologous sequence in the supercontig database (N terminus on supercontig_0011013: tBLASTn E = 8.1e-27, C terminus on supercontig_0033730: tBLASTn E = 2.3e-30) suggesting the orthologous gene exists elsewhere in the *H. contortus* genome. This gene appears to be expressed as RNA-seq reads map to its coding sequence on both supercontigs. *hc-13c1-1* is SL1 trans-spliced.

**Figure 3:**
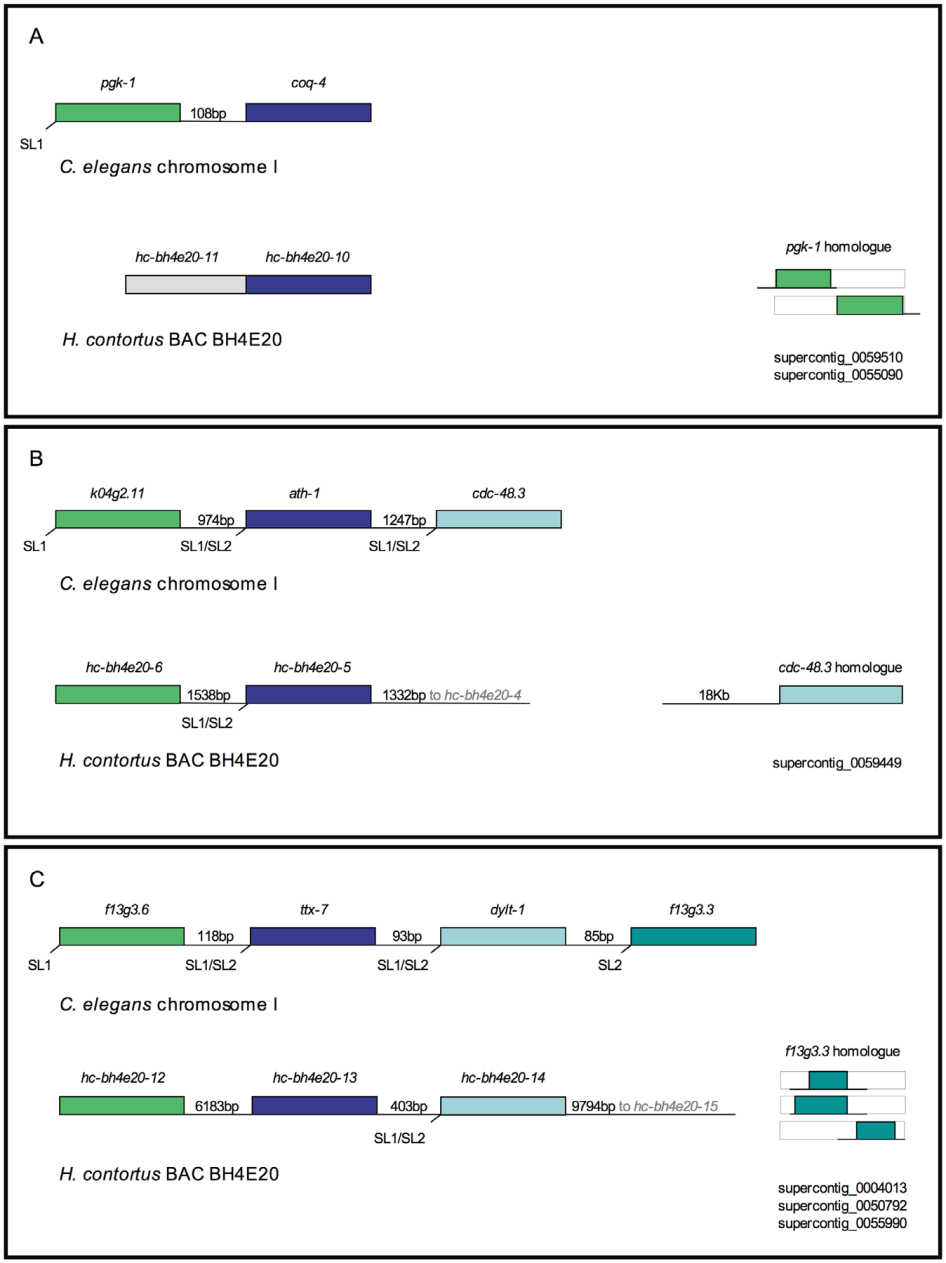
*H. contortus* orthologues of genes in operons in *C. elegans*. A. *C. elegans coq-4* is transcribed in an operon with upstream gene *pgk-1*. An *H. contortus* orthologue for *coq-4* but not *for pgk-1* was identified on BAC BH4E20. However, homologous sequence to *pgk-1* was identified in the supercontig database (supercontig_0059510: tBLASTn E = 4e-30, supercontig_0055090: tBLASTn E = 4. 5e-35), suggesting the orthologue exists elsewhere in the genome. The putative *H. contortus* orthologues of both *coq-4* (*hc-bh4e20-10*) and *pgk-1* are expressed, as shown by a large number of RNA-seq reads mapping to their loci. B. *ath-1* is transcribed in the middle of a three-gene operon in *C. elegans* with *k04g2.11* upstream and *cdc-48.3* downstream. *ath-1* and *k04g2.11* orthologues, *hc-bh4e20-5* and *hc-bh4e20-6*, were identified on *H. contortus* BAC BH4E20. The intergenic region between the parasite genes was 1538 bp. An orthologue of the downstream gene *cdc-48.3* was not identified on the sequence studied, but was on supercontig_0059449 (tBLASTn E = 7.5e-84). RNA-seq data suggested it was highly expressed and no upstream gene was identified in the available 18 kb. No SL-trimmed reads mapped to *hc-bh4e20-6*, but both SL1 and SL2 reads mapped to *hc-bh4e20-5*, consistent with it being a downstream gene in an operon. C. *C. elegans f13g3.6*, *ttx-7* and *dylt-1* are transcribed in a four-gene operon with downstream gene *f13g3.3*. The orthologues of *f13g3.6*, *ttx-1* and *dylt-1* were collinear on BAC BH4E20, but an orthologue of *f13g3.3* was not identified. However, sequence with homology to the F13G3.3 polypeptide was present in the *H. contortus* supercontig database (supercontig_0004013 tBLASTn E = 6.7e-23, supercontig_0050792 tBLASTn E = 4.3e-17, supercontig_0055990 tBLASTn E = 6.2e-12), although no upstream sequence was available for analysis. No RNA-seq reads mapped to the sequence with homology to *f13g3.3* on the three supercontigs, so this gene may not be expressed in *H. contortus*. Both SL1 and SL2 reads mapped to *hc-bh4e20-14*, consistent with it being a downstream gene in an operon.

Of the 12 *H. contortus* genes on BAC BH4E20 that have *C. elegans* orthologues on chromosome I, six of the *C. elegans* genes are known to be in operons: *coq-4* in CEOP1124, *k04g2.11* and *ath-1* in CEOP1449 and *f13g3.6*, *ttx-7*, *dylt-1* in CEOP1388. As shown in Figure 3, there seems to be partial conservation of these operons. Genes that are ‘missing’ from putative *H. contortus* orthologues of *C. elegans* operons can be identified elsewhere in the genome. Some of these genes appear to be expressed, including *H. contortus* orthologues of downstream genes in *C. elegans* operons, which lack their own promoter sequences in *C. elegans*.

### An HcRep sequence cluster is associated with a duplication breakpoint

**Figure 4:**
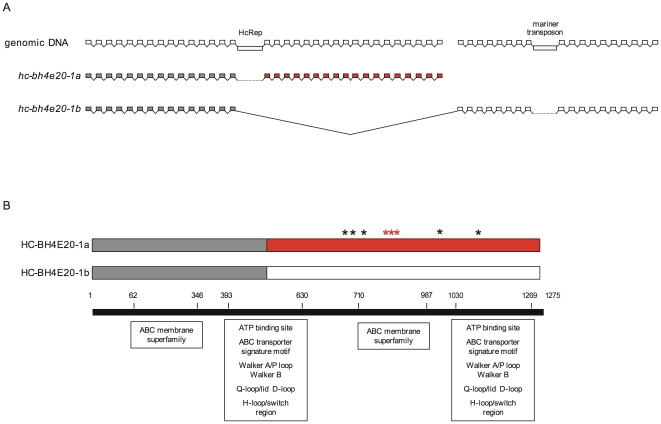
Possible alternative splicing of a P-glycoprotein. A. The last 36 exons of gene *hc-bh4e20-1*, a putative P-glycoprotein, appear to consist of a duplicated 18 exon region. The breakpoint is associated with an HcRep cluster. Intron and exon sizes are not to relative scale. B. The alternative 3′ ends of gene *hc-bh4e20-1* share 95% nucleotide identity, and the C-termini of the predicted polypeptides share 99% amino acid identity. Two of the five amino acid substitutions (black asterisks) lie within conserved domains and there is a three amino acid indel (red asterisks) within the ABC membrane superfamily domain of the C-terminus. Amino acid co-ordinates of the conserved domains are shown.

**Figure 5:**
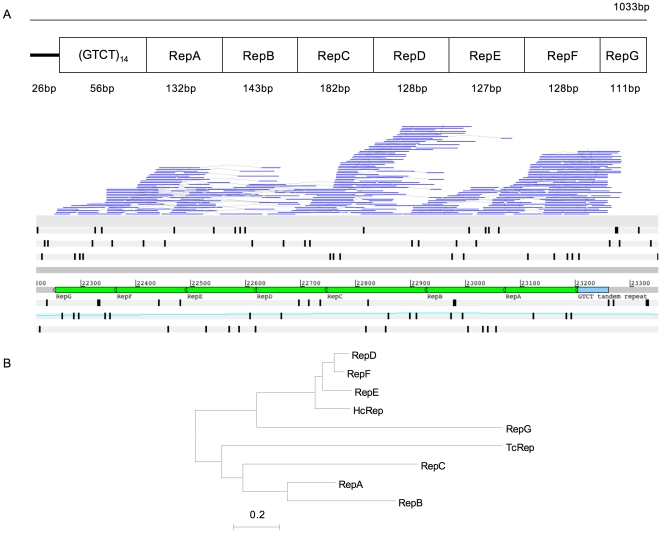
Seven copies of HcRep sequence identified on BAC BH4E20. A. Seven copies of the repeat element HcRep were identified on BAC BH4E20 and RNA-seq suggests they are expressed in the *H. contortus* genome. An Artemis screenshot shows the adjoining HcRep repeats (green), varying in length from 111 bp to 182 bp, and their association with a 56 bp GTCT tandem repeat (light blue). B. A neighbour-joining tree based on multiple sequence nucleotide alignment shows RepD, RepE, RepF and RepG share most homology with the published *H. contortus* HcRep consensus sequence (accession no. U86701), while RepA, RepB and RepC are more similar to *T. circumcincta* repeat element TcRep (accession no. M84610).

Characteristic repeat elements ‘HcRep’ and ‘TcRep’ have previously been described in the *H. contortus* and *T. circumcincta* genomes respectively [19], [20]. Seven copies of an HcRep-like sequence are present on BAC BH4E20, as an array in intron 15 of *hc-bh4e20-1a* (Figures 4 and 5). The first three copies (A–C) of the repeat element are most divergent, sharing more similarity to TcRep in *T. circumcincta* (accession number M84610) while copies D–G are more similar to the published HcRep consensus sequence (accession number U86701). The repeat elements are associated with an upstream (GTCT)_14_ tandem repeat. RNA-seq reads mapped to all seven copies of the repeat element, indicating HcRep-like sequences are expressed elements in the *H. contortus* genome. However, the expression level of these particular copies on BAC BH4E20 is not known since the RNA-seq reads may be mapping from expressed HcRep-like elements elsewhere in the genome. Previous studies have estimated over 0.1% of the *H. contortus* genome is related to HcRep1 based on Southern blot hybridisation intensity using the SE isolate [21]. A BLASTn search of the *H. contortus* supercontig database identified over 3000 matches (P<1e-05) to the published HcRep consensus sequence.

The gene within which the HcRep cluster is located, *hc-bh4e20-1a*, encodes a full-length P-glycoprotein, with the highest homology to *C. elegans* PGP-2 (Figure 4A and 4B). This gene has an interesting structure that suggests the HcRep cluster may be associated with a duplication break point. The last 36 exons of the gene, immediately downstream of the HcRep cluster, appear to consist of a duplicated 18 exon region. There is 99% amino acid identity between the putative translation products of the duplicated region, which appear to represent alternative splice variants of *hc-bh4e20-1a* to produce two different transcripts. There is also sequence with homology to a mariner transposase within the 23rd intron of the putative *hc-bh4e20-1b* spliced isoform.

### Mobile elements

Twelve putative transposable elements (TEs), eight of which contained transposon-associated conserved domains were identified on the two large contigs. Four putative polypeptides, sharing homology with the retrotransposon *rte-1* in *C. elegans*, had exonuclease endonuclease phosphatase, reverse transcriptase-like superfamily and non-long-terminal repeat retrotransposon and non-long terminal repeat retrovirus reverse transcriptase domains. Three putative polypeptides had conserved transposase-1 domains and one polypeptide had conserved pao retrotransposon peptidase and reverse transcriptase-like superfamily domains. Four transcripts shared most identity with TEs in other species but did not encode conserved domains (Figure 1, Figure 2 and Table S2).

RNA-seq reads mapped to nine of the 12 TEs. One of the remaining three, TE2, has no start methionine, so may represent a pseudogene. Although these results indicate that members of these TE families are transcriptionally active in the *H. contortus* genome, the extent to which these specific TE loci are transcribed is unclear. Inherent in the process of aligning whole transcriptome reads to only a portion of the genome, is the possibility that reads from a transcribed TE elsewhere in the genome could be mapped to a non-transcribed loci; in other words, reads from a functional TE could map to non-functional daughter progeny or to remnant sequence at ancient loci.

## Discussion

### Gene structure and content

*H. contortus* is a member of the closest phylogenetic clade of parasitic nematodes to *C. elegans* and the last common ancestor is estimated to have existed 400 million years ago [22]. Hence the extent to which genome structure and organisation is conserved between these two organisms is an important question. We have used a next generation transcriptomic approach to annotate two large contigs and these represent the largest contiguous sections of genomic sequence yet annotated for a strongylid nematode. One contig is known to be located on the X chromosome from previous genetic studies and this is supported by syntenic relationships of the annotated genes. The second contig is likely to be autosomal, being syntenic with *C. elegans* chromosome I.

The results of our annotations suggest gene size is consistently larger in *H. contortus* than in *C. elegans* with the average and median gene sizes being more than two-fold greater in the parasite than for orthologous genes in *C. elegans*. This is due to both a larger intron number and a larger intron length, with the mean spliced transcript size being very similar between the two species. This larger gene size is reflected in the gene density on the contigs, which is two to three times lower than occurs in *C. elegans*. The gene density on the X-linked contig is significantly less than on BAC BH4E20 consistent with a lower gene density on the X-chromosome than on the autosomes as is the case in *C. elegans* [10].

All annotated protein-coding genes, other than *hc-13c1-2*, had putative homologues in *C. elegans*. This represents 97.5% gene conservation, a significantly higher percentage than the ∼65% previously estimated with EST analysis [23]. The lowest BLASTp E value of 3e-08 recorded in this study corresponded to a BLASTx E value of 2e-09, compared to a BLASTx E value of 10e-5 to 10e-6 (bit score ≥50) in the EST analysis. So the higher figure for gene conservation in this study is unlikely to be explained by a lower stringency level for the identification of homologues, although BLAST E values will vary with the size and composition of the particular databases searched. It is also unlikely that the genes analysed in this work represent a more highly conserved subset than the EST data, but it is possible that homology is more likely to be detected for a survey of full-length genes than for clustered ESTs, as a number of the latter will represent only partial transcripts.

### Microsynteny and partial conservation of operons

Twelve genes on the *H. contortus* X-linked contig had convincing *C. elegans* orthologues, ten of which were located on the *C. elegans* X chromosome. Similarly, twelve genes on BAC BH4E20 had convincing *C. elegans* orthologues, eleven of which were located on *C. elegans* chromosome I. However, gene order was generally poorly conserved other than for a few gene clusters.

Although this pilot annotation covered only 590 kb of sequence, analysis of BAC end sequences suggest the pattern of a high level of conserved synteny but a low level of conserved gene order between the two species may be reflected throughout the rest of the genome. We identified 233 BACs for which both end sequences matched *C. elegans* genes and 50% of these pairs had matches on the same *C. elegans* chromosomes. These matches are not necessarily to orthologues since the full sequence of these genes are not available, so we have not undertaken extensive annotation and analysis. Nevertheless, this is significantly higher than the value expected by chance, suggesting a high level of syntenic conservation between the two species, consistent with the results for the two large contigs. These results are also consistent with studies comparing the *C. elegans* genome with those of *C. briggsae* and *B. malayi*, which detected high rates of rearrangement, with intra-chromosomal rearrangements more common than inter-chromosomal rearrangements [12], [24]–[27]. The rearrangement rate of *C. briggsae* has been estimated at 0.4–1 chromosomal breakages per Mb per million years [24], which is at least four times that of *D. melanogaster* [28]. Intra-chromosomal rearrangements are suggested to occur more commonly than inter-chromosomal rearrangements because they require fewer DNA breaks and because the conformation of the nuclear scaffold may maintain the association of local regions [26].

Although the overall gene order was relatively poorly conserved for the two large contigs, regions of conserved microsynteny between *H. contortus* and *C. elegans* were apparent. Four regions with collinear genes were identified, one on the X-linked contig and three on BAC BH4E20, two of which contained orthologues of genes in operons in *C. elegans*. These operons were only partially conserved since none of the *H. contortus* regions sharing synteny with *C. elegans* operons encoded the full complement of genes. Putative orthologues for all ‘missing’ genes could be identified elsewhere in the genome and RNA-seq reads mapped to all but one of these genes, suggesting they are expressed. It is unknown if these differences in operon structure represent gene gain to an operon in *C. elegans* or gene loss from an operon in *H. contortus* relative to the last common ancestor. The former perhaps seems more likely since once formed, operons are thought to be difficult to break, as downstream genes would be left without promoters [29]. However, breaking of operons is still possible; the 4% of operons that are not conserved between *C. elegans* and *C. briggsae* are a result of not only operon gains in *C. elegans* but also losses in *C. briggsae* [30]. For example, the same study identified a four-gene operon in *C. elegans* in which the first three genes were translocated to a different chromosome in *C. briggsae*. Despite this, all four genes were expressed in *C. briggsae* and the authors suggested the separated downstream gene had formed an operon with its new upstream gene. A similar mechanism may have facilitated expression of *hc-bh4e20-10* in *H. contortus*. This is a putative orthologue of *C. elegans coq-4*, a gene expressed downstream of *pgk-1* in two-gene operon CEOP1124. Despite *hc-bh4e20-10* lacking an upstream orthologue of *pgk-1*, it appears to be co-expressed with a different upstream gene, *hc-bh4e20-11*, which shares most homology with *f53a3.7* in *C. elegans*. *f53a3.7* is not in an operon in *C. elegans*, but it is possible that these genes have formed a new operon in *H. contortus*.

Functional constraints are thought to conserve intergenic regions within operons to approximately 100 bp in *C. elegans*, although increased intergenic distances have been reported in a small number of downstream genes trans-spliced with SL1 [12], [29], [31]. Operons with intergenic distances of greater than 100 bp (336 bp and 482 bp) have been identified in *B. malayi* [32], although this again may be facilitated by SL1 trans-splicing of downstream genes, since *B. malayi* lacks any SL2-like sequences [29]. In this study, intergenic distances of up to 6183 bp have been identified within the putative *H. contortus* operons and intergenic distances of up to 1538 bp have been identified preceding genes we have shown to be SL2 trans-spliced. Consistent with this, an operon encoding two genes, *Hco-des-2H* and *Hco-deg-3H*, with the latter SL2 trans-spliced, has been reported and the intergenic distance is ten times that between the orthologous pair of genes in *C. elegans* [16].

### Mobile elements and repetitive DNA

Approximately 12% of the *C. elegans* genome is comprised of TEs, although most of these are thought to be no longer mobile [10]. The 12 *H. contortus* TEs identified in this study represent just over 3% of the 590 kb genomic sequence analysed but the significant number of transcriptome reads mapping to TE loci suggests such elements may be highly active within the *H. contortus* genome.

Along with mutations generated by DNA-polymerase errors, TE insertions are the main internal drivers of genetic change, and the association of mobile elements with chromosome rearrangements in *Caenorhabditis* and *Drosophila* are well established [33]–[35]. An association of repetitive sequence with synteny break points in *Caenorhabditis* has also been identified [12], [36]. This may be an indirect association, if repetitive sequences represent derivatives of TEs or are generated by TE insertions, or a direct association if repetitive sequences induce ectopic recombination between repeats [36].

Both putative gene duplications identified in this study were associated with intronic transposon insertions, and one was also associated with the repeat element HcRep. Studies have suggested that TEs might be enriched within or flanking environmental response genes, such as the cytochrome P450 family [37]. Genomic plasticity would be predicted to be advantageous at these loci, as it would facilitate adaptive response to changes in the environment. Consistent with this hypothesis, a gene duplication associated with an intronic mariner transposon insertion and the repeat element HcRep was identified at a P-glycoprotein locus in this study. A TE insertion was observed in intron 5 of *hc-bh4e20-13*, which may also be an environmental response gene: the putative orthologue in *C. elegans*, *ttx-7*, encodes a myo-inositol monophosphatase required for normal thermotaxis and chemotaxis to sodium [38]. However, a retrotransposon-associated gene duplication was also identified involving the orthologue of *C. elegans folt-1*, a folate transporter, and intronic TE insertions were identified in two *H. contortus* genes more likely to have constitutive functions: orthologues of the *C. elegans mec-7* (a β-tubulin) and *air-2* (a serine/threonine protein kinase).

### Implications for the *H. contortus* genome project

This work indicates that the previous prediction of the genome size of *H. contortus* at 53 Mb may be an underestimate [39]. The *C. elegans* genome is 100 Mb and if the parasite genome has a similar gene complement, a gene density and size differing from the model worm by a two to three fold magnitude would be suggestive of a ∼200–300 Mb genome. This has obvious implications for the genome project, but may in part explain the current difficulties with assembly (Gilleard and Berriman, unpublished data).

Nematode genomes are evolving rapidly. Comparisons of *C. elegans* and *C. briggsae* genomes relative to mouse and human genomes (the former pair diverged ∼80–110 million years ago, the latter ∼75 million years ago), showed the nematodes have fewer 1∶1 orthologue pairs, more genes lacking matches in both species, a nearly three-fold higher nucleotide substitution rate and a dramatically higher chromosomal rearrangement rate [12]. *C. elegans* and *H. contortus* are estimated to have diverged 400 million years ago [21] and the impact of the *H. contortus* adaptation to parasitism on the (potentially highly plastic) nematode genome is unknown, but it was predicted that the retention of a conserved genetic core relating to essential biological processes would facilitate a comparative bioinformatic approach for gene discovery and annotation [2]. In this study, we found that 97.5% of genes had detectable homologues in *C. elegans* and 60% had clear orthologues. This is higher than anticipated from EST comparisons and suggests that comparative sequence analysis between the two species will be a powerful approach for gene annotation. Although global positional information of genes in *C. elegans* will be of limited use for finding orthologues in the parasite due to the high rate of intra-chromosomal rearrangements, homologous genes do appear to reside on the same chromosomes frequently, and where present, local regions of microsynteny can be used as supporting evidence of orthologous relationships.

In summary, the *H. contortus* transcriptome has been sequenced using Illumina technology and this pilot survey of 590 kb genomic sequence suggests RNA-seq will be a useful technique to annotate the *H. contortus* genome. The results also suggest the close phylogenetic relationship of *H. contortus* and *C. elegans* will also facilitate a comparative genomics approach, which will prove particularly useful for annotation of conserved genes with a low level of expression in the parasite.

## Materials and Methods

### *H. contortus* maintenance and culturing

All experimental procedures described in this manuscript were examined and approved by the Moredun Research Institute Experiments and Ethics Committee and were conducted under approved British Home Office licenses in accordance with the Animals (Scientific Procedures) Act of 1986. The Home Office license number is PPL 60/03899 and experimental IDs for these studies were E34/09 and E36/09. Experimental infections were performed by oral administration of 5000 L3 of the *H. contortus* MHco3 (ISE) isolate [15] into 4- to 9-month-old lambs that had been reared and maintained indoors under parasite-free conditions. 21-day-old adult worms were removed at post-mortem using an agar/mesh flotation method described in Jackson and Hoste [40] then rinsed and snap-frozen in liquid nitrogen and stored at −80°C.

### BAC library construction

Two BAC libraries were prepared by partially digesting PFGE plugs prepared from *H. contortus* larvae with Sau3AI or Apo1, respectively. High molecular weight DNA was resolved by CHEF gel electrophoresis and recovered by electroelution before ligating into BamHI- or EcoR1-cut pBACe3.6, respectively. The ligation was electroporated into DH10B cells (Invitrogen), plated, robotically picked and arrayed into 384 well microtitre plates before being replicated and tested for absence of bacterial and phage contamination.

### BAC sequencing and assembly

Seven BACs were chosen for sequencing from the 20,828 BAC end-sequences of the ongoing genome project and small insert libraries (2–4 kb) were constructed in *Sma* I digested pUC18. ABI PRISM BigDye Terminator (Applied Biosystems) forward and reverse plasmid end sequences were generated using an ABI3730 capillary sequencer and then assembled and manually finished using Phrap and Gap4, respectively, from the Staden package.

### Annotation and nomenclature

Two genomic sequences were annotated. The first consisted of a 409 kb contig, assembled as the consensus of five overlapping BAC insert sequences: haemapobac13c1, haemapobac7n11, haembac15g16, haembac18h7 and haembac18g2. Genetic analysis using microsatellite markers within this contig has shown it to be derived from the X chromosome [15]. The second sequence was a non-contiguous 181 kb BAC insert, BHA4E20Ge02.q2ky012. In this paper, these sequences are referred to as the X-linked contig and BAC BH4E20 respectively. All annotated genes are named ‘*hc*’ for *H. contortus*, followed by an identifier for the BAC insert sequence they are located on, followed by a number e.g. *hc-13c1-1*.

The subset of *H. contortus* putative orthologues of *C. elegans* genes were identified using the following criteria: the predicted polypeptide encoded by each putative orthologue had greater than 45% amino acid identity to a *C. elegans* protein, over greater than 80% of its length, and no sequence with higher identity to the *C. elegans* protein was present in the *H. contortus* shotgun assembly contig databases. Two neighbouring genes on the X-linked contig, *hc-13c1-5* and *hc-13c1-4*, were both homologous with *C. elegans folt-1* (50% and 48% amino acid identity, respectively), and may represent a recent duplication event. The structure of both *H. contortus* genes was essentially the same, so to avoid repetition in the comparison of gene structure between species, only *hc-13c1-5* was included in the analysis. Three genes were included in the subset of orthologues due to conserved microsynteny, although their amino acid identity was below the conservative cut-off. Distance trees were built from BLAST pairwise alignments of the top 100 hits in the NCBI non-redundant protein databases to each *H. contortus* polypeptide to confirm each parasite gene clustered with its putative *C. elegans* orthologue.

### cDNA library preparation and sequencing

Total RNA was isolated from a frozen pellet of 500 µl mixed sex adult worms using a standard Trizol (Invitrogen, 15596-026) protocol. The quality and quantity of the total RNA yield was assessed with a Bioanalyzer 2100 (Agilent). mRNA was isolated from 50 µg total RNA with magnetic beads (FastTrack MAG mRNA Isolation Kit, Invitrogen, K1580-01) according to the manufacturer's protocol and eluted in 35 µl RNase-free water. The mRNA was quantified with a NanoDrop 3300 Fluorospectrometer (Thermo Scientific). mRNA (0.5–1 µg) was fragmented with RNA Fragmentation Reagents (Ambion, AM8740): 31.5 µl mRNA was heated at 70°C with 3.5 µl 10× Fragmentation Buffer for 5 minutes, before adding 3.5 µl Stop Buffer. The fragmented mRNA was precipitated with 3.5 µl NaOAC pH 5.2, 2 µl glycogen and 100 µl 100% ethanol and incubated at −80°C for 30 minutes. Following centrifugation at 14000 rpm at room temperature for 15 minutes, the supernatant was removed and the pellet washed with 1 ml 70% ethanol in DEPC-treated water, then vortexed and centrifuged at 14000 rpm at room temperature for 10 minutes. The supernatant was removed and the pellet was air-dried for up to 30 minutes then re-suspended in 10.5 µl RNase-free water. First-strand and second-strand cDNA were synthesized according to the manufacturer's protocol (Superscript Double-stranded cDNA Synthesis Kit, Invitrogen) but with 3 µg/µl random hexamer primers (Invitrogen). The cDNA was cleaned using a QIAquick PCR Purification Kit (Qiagen).

Sequencing libraries for the Illumina GA II platform were constructed from the cDNA, with end-repair with Klenow polymerase, T4 DNA polymerase and T4 polynucleotide kinase (to blunt-end the DNA fragments). A single 3′ adenosine moiety was added to the cDNA using Klenow exo- and dATP. Illumina adapters were ligated onto the repaired ends of the cDNA and gel-electrophoresis was used to separate library DNA fragments from unligated adapters by selecting cDNA fragments between 200 and 250 bp in size. Ligated cDNA fragments were recovered following gel extraction and libraries were amplified by 18 cycles of PCR with Phusion DNA polymerase (Finnzymes Reagents).

The efficacy of each stage of library construction was ascertained in a quality control step that involved measuring the adapter-cDNA on an Agilent DNA 1000 chip. For each library, paired end 76 bp reads were produced from a single lane of an Illumina GA flowcell according to manufacturer's protocol.

### RNA-seq analysis

Transcriptomic reads were mapped to the reference genomic sequences using BWA (Burrows-Wheeler Aligner; [41]) and processed into BAM format using SAMtools [42]. Sorted and indexed BAM files were opened and viewed as stacks of paired reads over genomic sequence in Artemis [43], [44] (Figure S1).

To permit the identification of genes trans-spliced to SL1 and SL2, all transcriptomic reads containing published *H. contortus* SL1 or SL2 sequences (accession numbers Z69630 and AF215836 respectively) were extracted and the SL sequence was removed. There is a family of SL2 sequences in *C. elegans*, and the same may be true in *H. contortus*, so a single base pair mismatch from the published sequence was tolerated. The trimmed reads were aligned to the reference genomic sequences with BWA as two separate groups (depending on the SL sequence). The SL1 and SL2 BAM files were sorted and indexed with SAMtools and opened directly in Artemis to view the trimmed reads aligned to the annotated genomic sequences.

### Bioinformatics

Sequence similarity searches of predicted *H. contortus* genes and their conceptual translations were performed using BLAST at the National Centre for Biotechnology Information (www.ncbi.nlm.nih.gov/BLAST) and *C. elegans* Wormbase (www.wormbase.org/db/searches/blast_blat). The gene prediction software ‘Genefinder’ was run on the X-linked contig using *C. elegans* parameters (www.sanger.ac.uk/Projects/C_elegans/GENEFINDER). Conserved synteny was assessed by aligning the *H. contortus* X-linked contig and BAC BH4E20 with *C. elegans* chromosome X and chromosome I respectively, with the Artemis Comparison Tool (ACT) [45], [46].

For the analysis of BAC end-sequences, a BLASTx search of Wormpep with the 20,828 paired sequences (each end of 10,414 BAC insert sequences) in the *H. contortus* BAC end database was undertaken. The locus of the best-matched *C. elegans* gene for every hit with P<0.01 was recorded, before matching up each BAC end with its mate pair and comparing the loci of the putative homologues (Table S1).

## Supporting Information

Figure S1 **Annotation of the** ***H. contortus*** **genome with RNA-seq.** A typical Artemis screen shot with transcriptome reads from the highly expressed gene *hc-bh4e20.16* aligned to genomic sequence. Grey lines connect paired reads.
(TIF)

Table S1 ***H. contortus*** **BAC inserts with matches (P<0.01) to** ***C. elegans*** **proteins at each end.**
(DOC)

Table S2 **12 transposable elements identified in 590 kb genomic sequence.**
(DOC)
