# Supplementary figures and images for: Annotation of Two Large Contiguous Regions from the Haemonchus contortus Genome Using RNA-seq and Comparative Analysis with Caenorhabditis elegans
Source: PLoS One. 2011 Aug 15;6(8):e23216. doi: 10.1371/journal.pone.0023216 (PMC3156134; doi:10.1371/journal.pone.0023216)

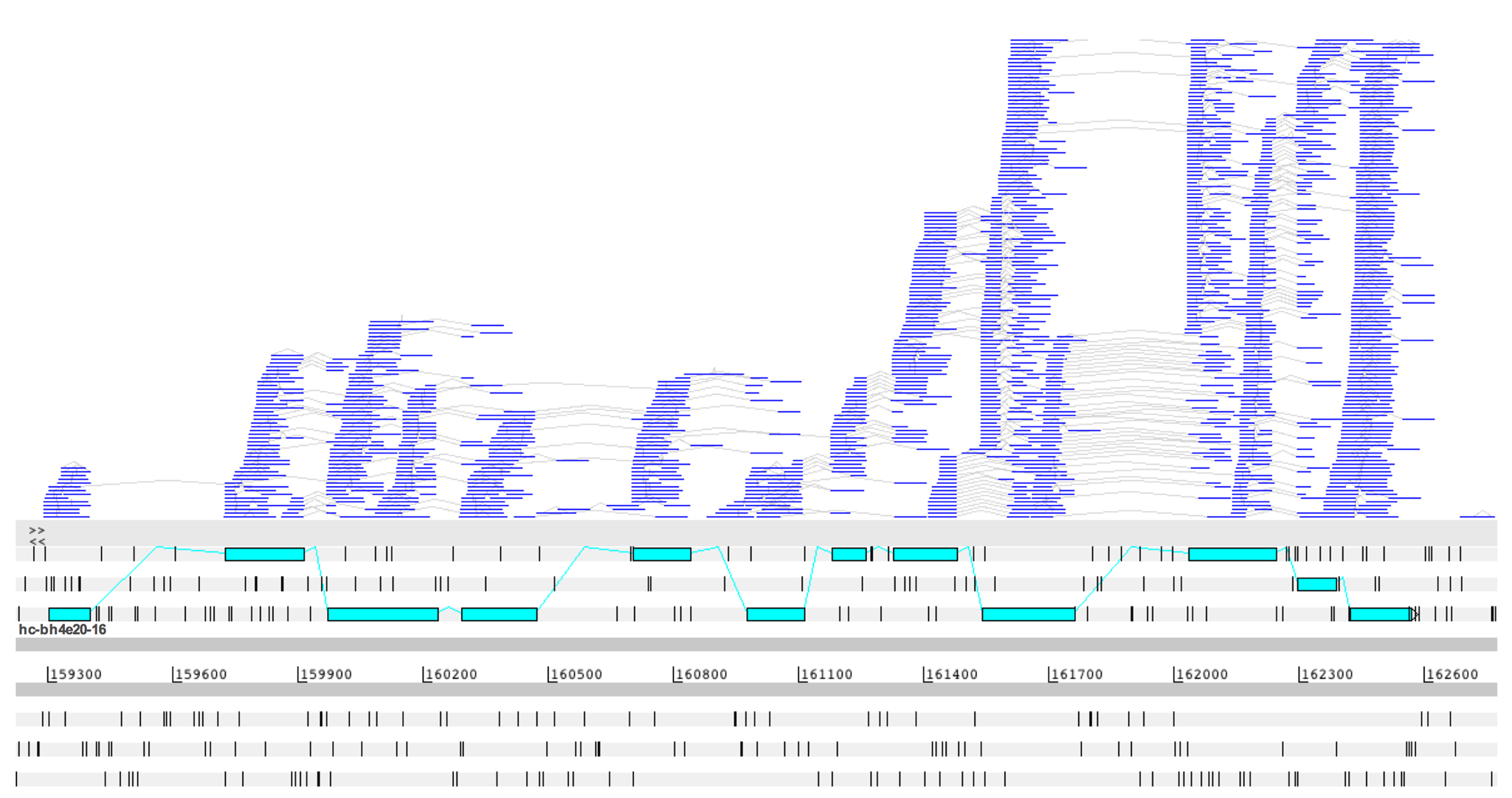

Supplement: Figure S1 — Annotation of the H. contortus genome with RNA-seq. A typical Artemis screen shot with transcriptome reads from the highly expressed gene hc-bh4e20.16 aligned to genomic sequence. Grey lines connect paired reads. (TIF) [file pone.0023216.s001.tif]
