# Supplementary material for: Annotation of Two Large Contiguous Regions from the Haemonchus contortus Genome Using RNA-seq and Comparative Analysis with Caenorhabditis elegans
Source: PLoS One. 2011 Aug 15;6(8):e23216. doi: 10.1371/journal.pone.0023216 (PMC3156134; doi:10.1371/journal.pone.0023216)
Supplement: Table S1 — H. contortus BAC inserts with matches (P<0.01) to C. elegans proteins at each end. (DOC) [file pone.0023216.s002.doc]

**Table S1. *H. contortus* BAC inserts with matches (P<0.01) to *C. elegans* proteins at each end.**

| ***H. contortus* BAC end** | ***C. elegans* polypeptide** | ***C. elegans* chromosome** |
| --- | --- | --- |
| haem_ends11i02.p1kSP6 | F25C8.3d | V |
| haem_ends11i02.q1kT7 | T09E8.1c | V |
| haem_ends11j22.p1kSP6 | K07C10.1 | II |
| haem_ends11j22.q1kT7 | T10H9.5c | V |
| haem_ends11k24.p1kSP6 | T10H9.5b | V |
| haem_ends11k24.q1kT7 | K07C10.1 | II |
| haem_ends11n16.p1kSP6 | C39D10.7 | X |
| haem_ends11n16.q1kT7 | D2085.1 | II |
| haem_ends11n19.p1kSP6 | F54E7.7 | III |
| haem_ends11n19.q1kT7 | F37C12.3 | III |
| haem_ends12b11.p1kSP6 | Y37E11AL.1 | IV |
| haem_ends12b11.q1kT7 | F54G2.2 | X |
| haem_ends12c24.p1kSP6 | F25F2.2a | III |
| haem_ends12c24.q1kT7 | T28H10.3 | V |
| haem_ends12g10.p1kSP6 | T01G1.1d | IV |
| haem_ends12g10.q1kT7 | T01G1.1c | IV |
| haem_ends12h13.p1kSP6 | ZK688.3 | III |
| haem_ends12h13.q1kT7 | B0496.3b | IV |
| haem_ends12i13.p1kSP6 | F57C12.5e | X |
| haem_ends12i13.q1kT7 | R07B1.10 | X |
| haem_ends12j14.p1kSP6 | Y51A2D.15 | V |
| haem_ends12j14.q1kT7 | K02F6.1 | II |
| haem_ends12j21.p1kSP6 | C55B7.9 | I |
| haem_ends12j21.q1kT7 | C41A3.1 | X |
| haem_ends12l12.p1kSP6 | CC8.1 | IV |
| haem_ends12l12.q1kT7 | K04B12.1 | II |
| haem_ends13b11.p1kSP6 | T05A10.5 | X |
| haem_ends13b11.q1kT7 | F01D5.5 | II |
| haem_ends13b13.p1kSP6 | C09D1.1b | I |
| haem_ends13b13.q1kT7 | C09D1.1g | I |
| haem_ends13d12.p1kSP6 | F08B4.2a | IV |
| haem_ends13d12.q1kT7 | F11A5.9 | V |
| haem_ends13e19.p1kSP6 | C26D10.4 | II |
| haem_ends13e19.q1kT7 | F09E5.5 | II |
| haem_ends13f07.p1kSP6 | T07G12.3 | IV |
| haem_ends13f07.q1kT7 | ZC132.6 | V |
| haem_ends13g02.p1kSP6 | F09E8.7b | IV |
| haem_ends13g02.q1kT7 | Y76B12C.2 | IV |
| haem_ends13h06.p1kSP6 | T06A4.1b | I |
| haem_ends13h06.q1kT7 | F29D11.1 | I |
| haem_ends13j22.p1kSP6 | C54G10.3 | V |
| haem_ends13j22.q1kT7 | T28B4.1c | X |
| haem_ends13l19.p1kSP6 | R12E2.3 | I |
| haem_ends13l19.q1kT7 | F58A6.4 | II |
| haem_ends14a18.p1kSP6 | F55B11.1 | IV |
| haem_ends14a18.q1kT7 | R05G6.6 | IV |
| haem_ends14b14.p1kSP6 | C52E4.5 | V |
| haem_ends14b14.q1kT7 | C17H12.5 | IV |
| haem_ends14b17.p1kSP6 | F55B11.1 | IV |
| haem_ends14b17.q1kT7 | R05G6.6 | IV |
| haem_ends14d11.p1kSP6 | W05E10.4 | V |
| haem_ends14d11.q1kT7 | T05A10.5 | X |
| haem_ends14e07.p1kSP6 | W09C3.8 | I |
| haem_ends14e07.q1kT7 | ZK632.1a | III |
| haem_ends14e08.p1kSP6 | W09C3.8 | I |
| haem_ends14e08.q1kT7 | ZK632.1a | III |
| haem_ends14e10.p1kSP6 | T08H4.1 | II |
| haem_ends14e10.q1kT7 | C03D6.3b | I |
| haem_ends14f19.p1kSP6 | T04B2.5 | IV |
| haem_ends14f19.q1kT7 | F58B4.1b | V |
| haem_ends14g05.p1kSP6 | ZC132.6 | V |
| haem_ends14g05.q1kT7 | Y61A9LA.10 | V |
| haem_ends14g07.p1kSP6 | F55F8.5 | I |
| haem_ends14g07.q1kT7 | Y71G12B.11b | I |
| haem_ends14g12.p1kSP6 | Y48G8AL.1 | I |
| haem_ends14g12.q1kT7 | W06H8.8g | V |
| haem_ends14g14.p1kSP6 | F35A5.4 | X |
| haem_ends14g14.q1kT7 | C05H8.1b | III |
| haem_ends14h09.p1kSP6 | F46C5.9 | II |
| haem_ends14h09.q1kT7 | C31C9.2 | II |
| haem_ends14h10.p1kSP6 | F42H10.3 | III |
| haem_ends14h10.q1kT7 | B0285.1c | III |
| haem_ends14k08.p1kSP6 | T04C9.1b | III |
| haem_ends14k08.q1kT7 | Y56A3A.27 | III |
| haem_ends14m03.p1kSP6 | F57F5.5 | V |
| haem_ends14m03.q1kT7 | F57F5.5 | V |
| haem_ends14p16.p1kSP6 | F18A1.7 | II |
| haem_ends14p16.q1kT7 | B0365.5a | V |
| haem_ends15a17.p1kSP6 | R02C2.7 | V |
| haem_ends15a17.q1kT7 | Y113G7B.12 | V |
| haem_ends15a20.p1kSP6 | Y106G6A.2a | I |
| haem_ends15a20.q1kT7 | F26E4.7a | I |
| haem_ends15b04.p1kSP6 | T22B11.1 | IV |
| haem_ends15b04.q1kT7 | F20G4.3 | I |
| haem_ends15c02.p1kSP6 | T20D3.11b | IV |
| haem_ends15c02.q1kT7 | C33A12.3 | IV |
| haem_ends15c19.p1kSP6 | Y104H12D.3 | IV |
| haem_ends15c19.q1kT7 | F36A4.6 | IV |
| haem_ends15d01.p1kSP6 | ZC513.8 | V |
| haem_ends15d01.q1kT7 | C34D4.15 | IV |
| haem_ends15f05.p1kSP6 | F55A12.3 | I |
| haem_ends15f05.q1kT7 | ZC123.4c | I |
| haem_ends15f08.p1kSP6 | F57C9.6 | I |
| haem_ends15f08.q1kT7 | R02D3.7 | IV |
| haem_ends15h03.p1kSP6 | H19N07.1 | V |
| haem_ends15h03.q1kT7 | Y42A5A.2 | V |
| haem_ends15j10.p1kSP6 | R02D3.7 | IV |
| haem_ends15j10.q1kT7 | Y73B6BL.3 | IV |
| haem_ends15l11.p1kSP6 | F36A2.3 | I |
| haem_ends15l11.q1kT7 | F25H2.1 | I |
| haem_ends16a20.p1kSP6 | ZK550.2 | IV |
| haem_ends16a20.q1kT7 | ZK550.2 | IV |
| haem_ends16d16.p1kSP6 | C10G8.4 | V |
| haem_ends16d16.q1kT7 | ZK131.7 | II |
| haem_ends16m14.p1kSP6 | T20D3.11b | IV |
| haem_ends16m14.q1kT7 | Y105C5B.10 | IV |
| haem_ends16n16.p1kSP6 | R151.10 | III |
| haem_ends16n16.q1kT7 | Y57G11C.31 | IV |
| haem_ends17b07.p1kSP6 | C42D4.8 | IV |
| haem_ends17b07.q1kT7 | C36E8.4 | III |
| haem_ends17c06.p1kSP6 | ZK1010.5 | III |
| haem_ends17c06.q1kT7 | ZK1010.5 | III |
| haem_ends17h20.p1kSP6 | K09H11.1 | V |
| haem_ends17h20.q1kT7 | T03D8.6b | V |
| haem_ends17k20.p1kSP6 | Y73B6BR.1b | IV |
| haem_ends17k20.q1kT7 | T01H10.2 | X |
| haem_ends17n09.p1kSP6 | F49E8.3b | IV |
| haem_ends17n09.q1kT7 | H06O01.3 | I |
| haem_ends18a20.p1kSP6 | Y54F10AM.8 | III |
| haem_ends18a20.q1kT7 | ZK783.1 | III |
| haem_ends18b16.p1kSP6 | H22K11.1 | X |
| haem_ends18b16.q1kT7 | H22K11.1 | X |
| haem_ends18c04.p1kSP6 | B0303.3 | III |
| haem_ends18c04.q1kT7 | C36E8.1 | III |
| haem_ends18c07.p1kSP6 | K09H9.2 | I |
| haem_ends18c07.q1kT7 | ZK512.11 | III |
| haem_ends18c20.p1kSP6 | F57B9.5 | III |
| haem_ends18c20.q1kT7 | C27F2.4 | III |
| haem_ends18e21.p1kSP6 | F11G11.10 | II |
| haem_ends18e21.q1kT7 | Y64G10A.7b | IV |
| haem_ends18h11.p1kSP6 | C49H3.4 | IV |
| haem_ends18h11.q1kT7 | C08F8.6 | IV |
| haem_ends18i23.p1kSP6 | C39H7.7 | IV |
| haem_ends18i23.q1kT7 | T19E7.5 | IV |
| haem_ends18j08.p1kSP6 | R07G3.2 | II |
| haem_ends18j08.q1kT7 | F48A11.1 | II |
| haem_ends18k15.p1kSP6 | ZK430.2 | II |
| haem_ends18k15.q1kT7 | W06F12.2c | III |
| haem_ends18m13.p1kSP6 | R09H10.4 | IV |
| haem_ends18m13.q1kT7 | Y17G7B.5a | II |
| haem_ends18o16.p1kSP6 | C39H7.7 | IV |
| haem_ends18o16.q1kT7 | T19E7.5 | IV |
| haem_ends18p05.p1kSP6 | F35H10.10 | IV |
| haem_ends18p05.q1kT7 | C02F4.2c | IV |
| haem_ends18p17.p1kSP6 | K03B8.2 | V |
| haem_ends18p17.q1kT7 | C52D10.12 | IV |
| haem_ends19a23.p1kSP6 | R06C1.3 | I |
| haem_ends19a23.q1kT7 | Y44E3B.2 | I |
| haem_ends19c22.p1kSP6 | F42A10.1 | III |
| haem_ends19c22.q1kT7 | F56D2.5 | III |
| haem_ends19e15.p1kSP6 | C14B9.8 | III |
| haem_ends19e15.q1kT7 | ZC132.4 | V |
| haem_ends19e19.p1kSP6 | Y48G9A.3 | III |
| haem_ends19e19.q1kT7 | Y111B2A.24 | III |
| haem_ends19f04.p1kSP6 | F49H12.5 | X |
| haem_ends19f04.q1kT7 | F22B7.7 | III |
| haem_ends19g19.p1kSP6 | E02C12.10 | V |
| haem_ends19g19.q1kT7 | ZK287.1 | V |
| haem_ends19l06.p1kSP6 | T09E8.2 | V |
| haem_ends19l06.q1kT7 | Y39B6A.14 | V |
| haem_ends19n10.p1kSP6 | W09C3.8 | I |
| haem_ends19n10.q1kT7 | F07G6.9 | X |
| haem_ends19o05.p1kSP6 | K09E2.1 | X |
| haem_ends19o05.q1kT7 | F13E6.6 | X |
| haem_ends19p15.p1kSP6 | F01D4.7 | IV |
| haem_ends19p15.q1kT7 | C47E12.3 | IV |
| haem_ends20a08.p1kSP6 | T16H12.2 | III |
| haem_ends20a08.q1kT7 | T10B10.7 | X |
| haem_ends20b08.p1kSP6 | T10H10.1 | X |
| haem_ends20b08.q1kT7 | C09E8.1a | II |
| haem_ends20c06.p1kSP6 | C18B12.3 | X |
| haem_ends20c06.q1kT7 | C18B12.6 | X |
| haem_ends20d09.p1kSP6 | F54E7.7 | III |
| haem_ends20d09.q1kT7 | F15A2.3 | X |
| haem_ends20d12.p1kSP6 | F46E10.10b | V |
| haem_ends20d12.q1kT7 | Y50D4C.4 | V |
| haem_ends20e23.p1kSP6 | Y55D5A.4 | III |
| haem_ends20e23.q1kT7 | F11A10.8 | IV |
| haem_ends20e24.p1kSP6 | C44H9.4 | V |
| haem_ends20e24.q1kT7 | C08H9.3a | II |
| haem_ends20i16.p1kSP6 | C29H12.2 | II |
| haem_ends20i16.q1kT7 | T05A8.5 | II |
| haem_ends20o05.p1kSP6 | Y113G7B.12 | V |
| haem_ends20o05.q1kT7 | F23C8.5 | I |
| haem_ends20p11.p1kSP6 | F35E2.5 | I |
| haem_ends20p11.q1kT7 | ZK250.9 | II |
| haem_ends5a05.p1kSP6 | F31C3.3 | I |
| haem_ends5a05.q1kT7 | K05C4.2 | I |
| haem_ends5b23.p1kSP6 | ZC247.1 | I |
| haem_ends5b23.q1kT7 | ZC247.1 | I |
| haem_ends5d08.p1kSP6 | W09C3.8 | I |
| haem_ends5d08.q1kT7 | ZK973.6 | I |
| haem_ends5d11.p1kSP6 | C09D1.1b | I |
| haem_ends5d11.q1kT7 | C09D1.1g | I |
| haem_ends5e11.p1kSP6 | W09C3.8 | I |
| haem_ends5e11.q1kT7 | Y38H6C.5 | V |
| haem_ends5j17.p1kSP6 | R08B4.1b | X |
| haem_ends5j17.q1kT7 | F52E4.1a | X |
| haem_ends5j19.p1kSP6 | F11C7.4 | X |
| haem_ends5j19.q1kT7 | T04F8.4 | X |
| haem_ends5l08.p1kSP6 | M04B2.1 | IV |
| haem_ends5l08.q1kT7 | Y70G10A.2 | III |
| haem_ends5m01.p1kSP6 | K04H4.2c | III |
| haem_ends5m01.q1kT7 | C27F2.2b | III |
| haem_ends5p02.p1kSP6 | ZC132.6 | V |
| haem_ends5p02.q1kT7 | F40D4.13 | V |
| haem_ends5p08.p1kSP6 | Y6D1A.1 | II |
| haem_ends5p08.q1kT7 | H22K11.1 | X |
| haem_ends6a11.p1kSP6 | C41A3.1 | X |
| haem_ends6a11.q1kT7 | ZC434.9b | I |
| haem_ends6b14.p1kSP6 | ZC410.4a | IV |
| haem_ends6b14.q1kT7 | T22B11.5 | IV |
| haem_ends6b15.p1kSP6 | T07H8.4a | V |
| haem_ends6b15.q1kT7 | Y105C5B.5 | IV |
| haem_ends6e19.p1kSP6 | K07D4.9 | II |
| haem_ends6e19.q1kT7 | T24H10.3 | II |
| haem_ends6e23.p1kSP6 | Y54G11A.8a | II |
| haem_ends6e23.q1kT7 | F22B5.1 | II |
| haem_ends6h06.p1kSP6 | ZC132.6 | V |
| haem_ends6h06.q1kT7 | R02D3.5 | IV |
| haem_ends6h21.p1kSP6 | ZC132.4 | V |
| haem_ends6h21.q1kT7 | Y39A1A.15c | III |
| haem_ends6i07.p1kSP6 | Y75B8A.31 | III |
| haem_ends6i07.q1kT7 | K01D12.12 | V |
| haem_ends6i09.p1kSP6 | T10H10.1 | X |
| haem_ends6i09.q1kT7 | T19D12.6 | II |
| haem_ends6j06.p1kSP6 | F31C3.3 | I |
| haem_ends6j06.q1kT7 | K05C4.2 | I |
| haem_ends6k09.p1kSP6 | ZC15.7 | V |
| haem_ends6k09.q1kT7 | F53B7.3 | V |
| haem_ends6l14.p1kSP6 | Y71A12B.9 | I |
| haem_ends6l14.q1kT7 | Y69A2AR.20 | IV |
| haem_ends6m18.p1kSP6 | F10G7.1 | II |
| haem_ends6m18.q1kT7 | 2RSSE.1 | II |
| haem_ends6p13.p1kSP6 | F25D1.2 | V |
| haem_ends6p13.q1kT7 | F53F8.5 | V |
| haem_ends7b22.p1kSP6 | ZK792.5 | IV |
| haem_ends7b22.q1kT7 | T12A7.1 | IV |
| haem_ends7c24.p1kSP6 | K12D9.1 | V |
| haem_ends7c24.q1kT7 | F27E11.3b | V |
| haem_ends7d07.p1kSP6 | F58E10.4 | V |
| haem_ends7d07.q1kT7 | W09C3.8 | I |
| haem_ends7e18.p1kSP6 | F46C5.3 | II |
| haem_ends7e18.q1kT7 | Y55F3AM.1 | IV |
| haem_ends7g06.p1kSP6 | T24C12.3 | X |
| haem_ends7g06.q1kT7 | ZK131.9 | II |
| haem_ends7g17.p1kSP6 | R07B7.10 | V |
| haem_ends7g17.q1kT7 | H22D07.1 | V |
| haem_ends7g20.p1kSP6 | ZK836.1 | V |
| haem_ends7g20.q1kT7 | W03A3.2 | III |
| haem_ends7j15.p1kSP6 | Y48G1C.7 | I |
| haem_ends7j15.q1kT7 | T01A4.1d | I |
| haem_ends7j18.p1kSP6 | ZK328.2 | III |
| haem_ends7j18.q1kT7 | T21C12.1a | III |
| haem_ends7m16.p1kSP6 | F42C5.6 | IV |
| haem_ends7m16.q1kT7 | F26A3.4 | I |
| haem_ends7m24.p1kSP6 | C32F10.2 | I |
| haem_ends7m24.q1kT7 | C32F10.2 | I |
| haem_ends8a14.p1kSP6 | F53C3.12 | II |
| haem_ends8a14.q1kT7 | T05A10.5 | X |
| haem_ends8c06.p1kSP6 | F07C3.8 | V |
| haem_ends8c06.q1kT7 | T01H8.5e | I |
| haem_ends8d15.p1kSP6 | Y47G6A.15 | I |
| haem_ends8d15.q1kT7 | ZC132.4 | V |
| haem_ends8d16.p1kSP6 | B0491.8c | II |
| haem_ends8d16.q1kT7 | M195.3 | II |
| haem_ends8d18.p1kSP6 | C24H11.9 | III |
| haem_ends8d18.q1kT7 | Y75B8A.24 | III |
| haem_ends8e15.p1kSP6 | F55A8.2c | IV |
| haem_ends8e15.q1kT7 | K07A9.2 | IV |
| haem_ends8f04.p1kSP6 | F36H9.6 | V |
| haem_ends8f04.q1kT7 | M28.1 | II |
| haem_ends8k23.p1kSP6 | C02H7.3a | X |
| haem_ends8k23.q1kT7 | B0511.5 | I |
| haem_ends9a22.p1kSP6 | F58B4.1b | V |
| haem_ends9a22.q1kT7 | Y105C5B.5 | IV |
| haem_ends9e10.p1kSP6 | C08E3.6 | II |
| haem_ends9e10.q1kT7 | H10E21.2 | III |
| haem_ends9g10.p1kSP6 | ZK637.13 | III |
| haem_ends9g10.q1kT7 | B0348.4b | V |
| haem_ends9h24.p1kSP6 | R05F9.12 | II |
| haem_ends9h24.q1kT7 | R05F9.12 | II |
| haem_ends9i15.p1kSP6 | Y113G7B.12 | V |
| haem_ends9i15.q1kT7 | C39H7.7 | IV |
| haem_ends9j18.p1kSP6 | F42C5.8 | IV |
| haem_ends9j18.q1kT7 | T25G3.4 | I |
| haem_ends9n15.p1kSP6 | F59B2.2 | III |
| haem_ends9n15.q1kT7 | Y105E8A.13 | I |
| haem_ends9p06.p1kSP6 | F48E8.1c | III |
| haem_ends9p06.q1kT7 | Y55D5A.3 | III |
| haem1g11.p1kSP6 | F21A10.4 | X |
| haem1g11.q1kT7 | C52D10.12 | IV |
| haem1g23.p1kSP6 | T02G5.7 | II |
| haem1g23.q1kT7 | Y17G7B.10b | II |
| HaemApoBac_10i19.p1kSP6 | R11A5.1b | I |
| HaemApoBac_10i19.q1kT7 | Y48G8AL.5 | I |
| HaemApoBac_10n01.p1kSP6 | ZK858.1 | I |
| HaemApoBac_10n01.q1kT7 | T09B4.9 | I |
| HaemApoBac_11d23.p1kSP6 | Y111B2A.20 | III |
| HaemApoBac_11d23.q1kT7 | F49D11.4 | I |
| HaemApoBac_11g14.p1kSP6 | D1044.8 | III |
| HaemApoBac_11g14.q1kT7 | Y57G11C.5 | IV |
| HaemApoBac_11k10.p1kSP6 | K03H1.1 | III |
| HaemApoBac_11k10.q1kT7 | T06A4.3a | I |
| HaemApoBac_11m09.p1kSP6 | C02C6.1b | X |
| HaemApoBac_11m09.q1kT7 | ZK455.7 | X |
| HaemApoBac_11n24.p1kSP6 | C05D11.2 | III |
| HaemApoBac_11n24.q1kT7 | R09H3.1 | X |
| HaemApoBac_11o17.p1kSP6 | ZK892.4 | II |
| HaemApoBac_11o17.q1kT7 | Y67D8C.5 | IV |
| HaemApoBac_11o24.p1kSP6 | C05D11.2 | III |
| HaemApoBac_11o24.q1kT7 | Y87G2A.12 | I |
| HaemApoBac_12a19.p1kSP6 | F54G2.1a | X |
| HaemApoBac_12a19.q1kT7 | W09C3.8 | I |
| HaemApoBac_12m11.p1kSP6 | T08A9.11b | X |
| HaemApoBac_12m11.q1kT7 | F41C3.8b | II |
| HaemApoBac_12m12.p1kSP6 | F36A4.8 | IV |
| HaemApoBac_12m12.q1kT7 | F41C3.8b | II |
| HaemApoBac_13b10.p1kSP6 | W06H8.8g | V |
| HaemApoBac_13b10.q1kT7 | ZK863.8 | V |
| HaemApoBac_13c22.p1kSP6 | T24C12.3 | X |
| HaemApoBac_13c22.q1kT7 | ZK131.8 | II |
| HaemApoBac_13e22.p1kSP6 | D2013.5 | II |
| HaemApoBac_13e22.q1kT7 | T26C5.3a | II |
| HaemApoBac_13k20.p1kSP6 | W09B7.1 | V |
| HaemApoBac_13k20.q1kT7 | Y23H5A.7b | I |
| HaemApoBac_13n05.p1kSP6 | ZK250.9 | II |
| HaemApoBac_13n05.q1kT7 | ZK131.7 | II |
| HaemApoBac_13o08.p1kSP6 | F07C3.3 | V |
| HaemApoBac_13o08.q1kT7 | F15B10.1a | IV |
| HaemApoBac_13o15.p1kSP6 | C11D2.4 | IV |
| HaemApoBac_13o15.q1kT7 | C03A3.3 | X |
| HaemApoBac_13o16.p1kSP6 | C11D2.4 | IV |
| HaemApoBac_13o16.q1kT7 | C03A3.3 | X |
| HaemApoBac_13o19.p1kSP6 | F52H3.2 | II |
| HaemApoBac_13o19.q1kT7 | F26G1.6 | II |
| HaemApoBac_14b04.p1kSP6 | F48F7.2 | X |
| HaemApoBac_14b04.q1kT7 | F08B12.1 | X |
| HaemApoBac_14b20.p1kSP6 | K06A5.6 | I |
| HaemApoBac_14b20.q1kT7 | F08H9.5 | V |
| HaemApoBac_14i03.p1kSP6 | F48F7.2 | X |
| HaemApoBac_14i03.q1kT7 | F08B12.1 | X |
| HaemApoBac_15d16.p1kSP6 | Y55F3AL.1 | IV |
| HaemApoBac_15d16.q1kT7 | C06G8.1 | IV |
| HaemApoBac_15e06.p1kSP6 | ZK675.3a | II |
| HaemApoBac_15e06.q1kT7 | F02E11.1 | II |
| HaemApoBac_15i19.p1kSP6 | K07A12.7 | I |
| HaemApoBac_15i19.q1kT7 | F55F8.1 | I |
| HaemApoBac_15k07.p1kSP6 | F22E12.1 | V |
| HaemApoBac_15k07.q1kT7 | Y46H3A.7 | V |
| HaemApoBac_15k15.p1kSP6 | Y55F3AL.1 | IV |
| HaemApoBac_15k15.q1kT7 | C06G8.1 | IV |
| HaemApoBac_15m16.p1kSP6 | K03F8.2 | III |
| HaemApoBac_15m16.q1kT7 | F25F2.2a | III |
| HaemApoBac_15m22.p1kSP6 | D1037.4 | I |
| HaemApoBac_15m22.q1kT7 | F10D11.6 | I |
| HaemApoBac_16f04.q1kT7 | C24H11.4 | III |
| HaemApoBac_16f14.p1kSP6 | F30F8.5b | I |
| HaemApoBac_16g20.p1kSP6 | E01A2.7 | I |
| HaemApoBac_16g20.q1kT7 | ZK354.3 | IV |
| HaemApoBac_16i23.p1kSP6 | T21D12.9b | IV |
| HaemApoBac_16i23.q1kT7 | K01A6.2d | IV |
| HaemApoBac_16j24.p1kSP6 | W09G3.3 | I |
| HaemApoBac_16j24.q1kT7 | ZK973.6 | I |
| HaemApoBac_16m03.p1kSP6 | EEED8.9 | II |
| HaemApoBac_16m03.q1kT7 | EEED8.16 | II |
| HaemApoBac_17f06.p1kSP6 | H22K11.1 | X |
| HaemApoBac_17f06.q1kT7 | T05A12.3 | IV |
| HaemApoBac_17k07.p1kSP6 | R02D5.3 | V |
| HaemApoBac_17k07.q1kT7 | F11C7.3a | X |
| HaemApoBac_17l05.p1kSP6 | R13H9.1 | IV |
| HaemApoBac_17l05.q1kT7 | F25B3.3 | V |
| HaemApoBac_17m02.p1kSP6 | F56C9.10b | III |
| HaemApoBac_17m02.q1kT7 | K10H10.1 | II |
| HaemApoBac_17m03.p1kSP6 | F56C9.10b | III |
| HaemApoBac_17m03.q1kT7 | K10H10.1 | II |
| HaemApoBac_18e20.p1kSP6 | C11D2.6c | IV |
| HaemApoBac_18e20.q1kT7 | F18F11.4 | IV |
| HaemApoBac_18i04.p1kSP6 | K07C5.6 | V |
| HaemApoBac_18i04.q1kT7 | F21C10.7 | V |
| HaemApoBac_18l21.p1kSP6 | F19H8.4 | II |
| HaemApoBac_18l21.q1kT7 | F14E5.2b | II |
| HaemApoBac_18m24.p1kSP6 | C45E1.1b | I |
| HaemApoBac_18m24.q1kT7 | K10D3.2 | I |
| HaemApoBac_1e03.p1kSP6 | Y54H5A.1 | III |
| HaemApoBac_1e03.q1kT7 | H19M22.2d | III |
| HaemApoBac_1i16.p1kSP6 | ZK20.4a | II |
| HaemApoBac_1i16.q1kT7 | Y79H2A.3a | III |
| HaemApoBac_1k18.p1kSP6 | F09B12.3 | X |
| HaemApoBac_1k18.q1kT7 | C05D9.5 | X |
| HaemApoBac_1m07.p1kSP6 | C17G1.3b | X |
| HaemApoBac_1m07.q1kT7 | Y37E11AR.5 | IV |
| HaemApoBac_1n06.p1kSP6 | C17G1.3b | X |
| HaemApoBac_1n06.q1kT7 | Y37E11AR.5 | IV |
| HaemApoBac_2d01.p1kSP6 | ZK1128.7 | III |
| HaemApoBac_2d01.q1kT7 | T04A8.14 | III |
| HaemApoBac_2m17.p1kSP6 | H14E04.5 | III |
| HaemApoBac_2m17.q1kT7 | Y40B1A.4 | I |
| HaemApoBac_2m18.p1kSP6 | H14E04.5 | III |
| HaemApoBac_2m18.q1kT7 | Y40B1A.4 | I |
| HaemApoBac_3l16.p1kSP6 | K08E5.3a | III |
| HaemApoBac_3l16.q1kT7 | F18E2.3 | V |
| HaemApoBac_3l17.p1kSP6 | Y43F8C.13 | V |
| HaemApoBac_3l17.q1kT7 | T05C7.1 | IV |
| HaemApoBac_4f02.p1kSP6 | B0286.2a | II |
| HaemApoBac_4f02.q1kT7 | C55B7.3 | I |
| HaemApoBac_4f04.p1kSP6 | B0286.2a | II |
| HaemApoBac_4f04.q1kT7 | C55B7.3 | I |
| HaemApoBac_4g16.p1kSP6 | F58B4.1b | V |
| HaemApoBac_4g16.q1kT7 | F13E6.6 | X |
| HaemApoBac_4g19.p1kSP6 | T20G5.1 | III |
| HaemApoBac_4g19.q1kT7 | Y47D3B.7 | III |
| HaemApoBac_4h04.p1kSP6 | BE0003N10.2 | III |
| HaemApoBac_4h04.q1kT7 | Y55B1BR.4 | III |
| HaemApoBac_4h14.p1kSP6 | F55F8.5 | I |
| HaemApoBac_4h14.q1kT7 | Y71G12B.11b | I |
| HaemApoBac_4k16.p1kSP6 | T25B9.9 | IV |
| HaemApoBac_4k16.q1kT7 | F33H12.6 | II |
| HaemApoBac_4l15.p1kSP6 | T25B9.9 | IV |
| HaemApoBac_4l15.q1kT7 | ZK250.9 | II |
| HaemApoBac_4l16.p1kSP6 | T25B9.9 | IV |
| HaemApoBac_4l16.q1kT7 | F59H6.5 | II |
| HaemApoBac_4n06.p1kSP6 | T09E8.1f | V |
| HaemApoBac_4n06.q1kT7 | B0222.3 | V |
| HaemApoBac_4p07.p1kSP6 | ZK430.2 | II |
| HaemApoBac_4p07.q1kT7 | B0286.2a | II |
| HaemApoBac_5b17.p1kSP6 | R05A10.5 | IV |
| HaemApoBac_5b17.q1kT7 | ZK270.1 | I |
| HaemApoBac_5e08.p1kSP6 | Y49A3A.2 | V |
| HaemApoBac_5e08.q1kT7 | T05C12.10 | II |
| HaemApoBac_5g18.p1kSP6 | D2024.3 | IV |
| HaemApoBac_5g18.q1kT7 | C02F4.2c | IV |
| HaemApoBac_5j04.p1kSP6 | F16F9.4 | X |
| HaemApoBac_5j04.q1kT7 | T05A10.5 | X |
| HaemApoBac_5k18.p1kSP6 | F53A3.2 | III |
| HaemApoBac_5k18.q1kT7 | F59A2.6 | III |
| HaemApoBac_5n03.p1kSP6 | F52C12.4 | IV |
| HaemApoBac_5n03.q1kT7 | ZK617.1b | IV |
| HaemApoBac_6c06.p1kSP6 | Y113G7B.12 | V |
| HaemApoBac_6c06.q1kT7 | F26B1.3 | I |
| HaemApoBac_6e02.p1kSP6 | T15B7.1 | V |
| HaemApoBac_6e02.q1kT7 | T04D3.3b | I |
| HaemApoBac_6n13.p1kSP6 | K10H10.1 | II |
| HaemApoBac_6n13.q1kT7 | W02G9.2 | V |
| HaemApoBac_7a08.p1kSP6 | C04G6.3 | II |
| HaemApoBac_7a08.q1kT7 | ZK131.8 | II |
| HaemApoBac_7m17.p1kSP6 | T03D8.5 | V |
| HaemApoBac_7m17.q1kT7 | T03D8.5 | V |
| HaemApoBac_8e23.p1kSP6 | F22B3.9 | IV |
| HaemApoBac_8e23.q1kT7 | T04A8.7a | III |
| HaemApoBac_8e24.p1kSP6 | F22B3.9 | IV |
| HaemApoBac_8e24.q1kT7 | T04A8.7a | III |
| HaemApoBac_8g05.p1kSP6 | T19A6.4 | I |
| HaemApoBac_8g05.q1kT7 | C37A2.2 | I |
| HaemApoBac_8g06.p1kSP6 | T19A6.4 | I |
| HaemApoBac_8g06.q1kT7 | C37A2.2 | I |
| HaemApoBac_8h13.p1kSP6 | C52E12.2b | II |
| HaemApoBac_8h13.q1kT7 | F54B3.3 | II |
| HaemApoBac_8l02.p1kSP6 | W03D2.1c | IV |
| HaemApoBac_8l02.q1kT7 | F49D11.4 | I |
| HaemApoBac_9b23.p1kSP6 | D2092.1b | I |
| HaemApoBac_9b23.q1kT7 | DY3.2 | I |
| HaemApoBac_9o07.p1kSP6 | Y110A7A.6a | I |
| HaemApoBac_9o07.q1kT7 | ZC132.6 | V |
| HaemApoBac_9p03.p1kSP6 | Y38C1AB.5 | IV |
| HaemApoBac_9p03.q1kT7 | Y17G7B.1 | II |

233 *H. contortus* BAC end mate pairs with the best-matched *C. elegans* proteins (BLASTx search of Wormpep, P<0.01) and the *C. elegans* gene loci. 118 BAC end pairs (50.64%) hit *C. elegans* genes on the same chromosome compared to 16.67% that would be expected by chance if there was no conservation of chromosomal location.
