# Supplementary material for: Annotation of Two Large Contiguous Regions from the Haemonchus contortus Genome Using RNA-seq and Comparative Analysis with Caenorhabditis elegans
Source: PLoS One. 2011 Aug 15;6(8):e23216. doi: 10.1371/journal.pone.0023216 (PMC3156134; doi:10.1371/journal.pone.0023216)
Supplement: Table S2 — 12 transposable elements identified in 590 kb genomic sequence. (DOC) [file pone.0023216.s003.doc]

**Table S2. 12 transposable elements identified in 590 kb genomic sequence.**

| **Transposable Element** | **Locus (bp)** | **Nearest gene** | **Conserved Domains** |
| --- | --- | --- | --- |
| TE1 | X-linked contig: 215709-217648 | intron 10 of *hc-18h7-1* | transposase-1 |
| TE2 | X-linked contig: 312635-313513 | - | - |
| TE3 | X-linked contig: 356450-359237 | - | Pao retrotransposon peptidase, reverse transcriptase-like |
| TE4 | X-linked contig: 368455-371726 | 5' end of *hc-18g2-4* | reverse transcriptase, retrotransposon, DYN1, exo/endonuclease phosphatase |
| TE5 | X-linked contig: 400308-401276 | - | transposase-1 |
| TE6 | X-linked contig: 197502-197960 | - | - |
| TE7 | X-linked contig: 181445-181903 | - | - |
| TE8 | X-linked contig: 60349-63330 | intron 6 of *hc-13c1-5* | reverse transcriptase, retrotransposon, exo/endonuclease phosphatase |
| TE9 | BAC BH4E20: 5059-6003 | intron 23 of *hc-bh4e20-1b* | transposase-1 |
| TE10 | BAC BH4E20: 96027-98960 | intron 6 of *hc-bh4e20-7* | exo/endonuclease phosphatase, non-LTR retrotransposon, retrovirus reverse transcriptase |
| TE11 | BAC BH4E20: 131543-131938 | intron 5 of *hc-bh4e20-13* | - |
| TE12 | BAC BH4E20: 166085-168694 | 3' end of *hc-bh4e20-16* | reverse transcriptase |

The loci of 12 putative transposable elements (TEs) in the X-linked contig and BAC BH4E20, with the nearest annotated gene to each.
